# Mothers of children with disabilities: exploring lived experiences, challenges, and divorce risk

**DOI:** 10.3389/fpsyg.2024.1399419

**Published:** 2024-11-08

**Authors:** Yasir A. Alsamiri, Ayesha A. Alaghdaf, Ibraheem M. Alsawalem, Basheer A. Allouash, Seham D. Alfaidi

**Affiliations:** ^1^Department of Education, Islamic University of Madinah, Madinah City, Saudi Arabia; ^2^Department of Psychology, University of Hail, Hail City, Saudi Arabia; ^3^Department of Special Education, University of Hail, Hail City, Saudi Arabia; ^4^Department of Social Work, University of Hail, Hail City, Saudi Arabia; ^5^Department of Psychology, Hafr Al Batin University, Hafar Al Batin, Saudi Arabia

**Keywords:** divorce, stigma, mental health, Islamic faith, children with disability

## Abstract

This qualitative study explores the psychological challenges faced by divorced Saudi mothers who are raising children with disabilities. The study highlights the impact of having a child with a disability on marital stability and family issues, and the psychological challenges experienced by single mothers. Using semi-structured in-depth interviews, data were collected from five divorced mothers recruited from a private daycare center. Participants ranged in age from 34 to 53, each with one child with a disability, and an average divorce period of four years. The thematic analysis revealed that single/divorced Saudi mothers of children with disabilities experienced significant psychological strain characterized by fear, shock and attempts at religious acceptance to cope with their child’s condition. Additionally, these mothers reported facing substantial social challenges, including societal ridicule and limited remarriage prospects, highlighting the broader impact of having a child with a disability on their lives. The study underscores the need for tailored psychological support and interventions for families of children with disabilities, especially for mothers who are significantly impacted by the challenges associated with their child’s condition. Additionally, the study emphasizes the negative impact of societal attitudes towards disability, which can contribute to social problems and psychological distress for families. The study’s findings suggest the need for specialized clinics and support groups to help families cope with the unique challenges they face.

## 1 Introduction

Raising a child with a disability presents a unique set of challenges for any family. However, for divorced Saudi mothers, these difficulties can be compounded by social stigma, limited access to resources, and the weight of single parenthood. This research delves into the lived experiences of divorced Saudi mothers raising children with disabilities, with a specific focus on the psychological challenges that contribute to marital breakdown.

In Saudi culture, disability is deeply influenced by religious beliefs and societal norms, with mothers playing a crucial role in caring for children with disabilities (Madi et al., 2019). Mothers face challenges including community stigma, discrimination, and increased pressure during crises like COVID-19 (Madi et al., 2019; Hammad, 2021). They experience lower satisfaction in social and economic aspects of quality of life compared to mothers of children without disabilities (Alwhaibi et al., 2018). Mothers of children with autism spectrum disorder (ASD) encounter difficulties in obtaining diagnoses, accessing resources, and dealing with cultural stigma (Mursi and Sulaimani, 2022). Despite these challenges, Saudi mothers demonstrate resilience and attribute high importance to their children’s health and functioning (Alwhaibi et al., 2018). The studies emphasize the need for increased social support, professional help, and societal changes to alleviate the burden on mothers and improve their quality of life (Alwhaibi et al., 2018; Mursi and Sulaimani, 2022).

Literature suggests that the very presence of a child with a disability can be a risk factor for divorce. Existing literature consistently highlights the elevated levels of stress, anxiety, and depression experienced by parents of children with disabilities (Song et al., 2018; Gruszka et al., 2023). This study specifically focuses on mothers, who often act as primary caregivers, and explores how caring for a child with additional needs can exacerbate existing marital problems or create new ones (Haimour and Abu-Hawwash, 2012). The type and severity of the child’s disability can further strain the situation, as parents of children with conditions like autism may face more intense and constant emotional burdens (Salehi et al., 2017).

Studies have shown a correlation between having a child with a disability and a higher risk of divorce, particularly in Saudi Arabia (Al-Shammari and Al-Harbi, 2021; Al-Shammari, 2015). This increased strain can lead to a breakdown in communication, neglect of marital and family responsibilities, and ultimately, exacerbate existing psychological trauma within the family (Namkung et al., 2015).

Despite acknowledging the detrimental effects of divorce on families, a critical gap exists in research – a lack of qualitative analysis exploring the specific link between having a child with a disability and divorce rates (Daroni et al., 2018; Doherty, 2008). This gap underscores the need for in-depth qualitative research that delves deeper into the challenges faced by mothers in such situations (Anderson, 2014). Understanding the specific psychological challenges these mothers encounter is crucial, as their perspectives can inform targeted support systems and interventions.

The present study employs a qualitative research method to explore the perceptions and lived experiences of Saudi mothers of children with disabilities, with a focus on the psychological challenges that may contribute to divorce. Qualitative research is particularly well-suited for examining the subjective experiences and interpretations of individuals, providing a nuanced understanding of their thoughts, feelings, and motivations (Creswell et al., 2007; Ryder et al., 2019). Moreover, this approach allows for the discovery of unforeseen themes and challenges, offering valuable insights for future quantitative studies and intervention programs, ultimately highlighting the need for further investigation in this area. This approach aligns with the study’s aim to capture the psychological problems resulting from having a child with a disability that may lead to divorce.

The significance of this research extends beyond national borders, as it addresses a global issue with far-reaching implications. The study’s findings can contribute to a deeper understanding of the unique challenges faced by families of children with disabilities, particularly in the context of Saudi Arabian culture, where societal attitudes and norms play a crucial role in shaping the experiences of these families.

### 1.1 Research problem

The increased risk of divorce is associated with various negative consequences, such as its impact on psychological well-being, the breakdown of marital life, and the disintegration of families, ultimately affecting societal development. Al-Shammari and Al-Harbi (2021) found that in societies with high divorce rates, there is a corresponding increase in psychological problems within these families. Moreover, Al-Shammari (2015) observed a 75% increase in divorce rates among families in the Kingdom of Saudi Arabia that have a child with autism spectrum disorder or a disability in general, particularly affecting mothers who are significantly impacted upon discovering their child’s condition. This situation often leads to the neglect of marital and family responsibilities, contributing to psychological trauma within the family. Additionally, Namkung et al. (2015) confirmed that having a child with developmental disabilities may lead to divorce, influenced by factors such as family size and the child’s position in the birth order.

Furthermore, divorce has been shown to significantly impact the value system and structure of marital life, leading to family disintegration, especially among families of individuals with disabilities (Al-Maamari, 2015; Sharqi, 2018). It is noteworthy that foreign studies have highlighted the lack of qualitative analysis on the impact of having a child with a disability on the occurrence of divorce, emphasizing the need for a deeper understanding of the associated problems (Daroni et al., 2018; Doherty, 2008).

### 1.2 Research question

The current study aims to address the following primary inquiry: What are the psychological challenges stemming from raising a child with a disability that contribute to divorce, as perceived by mothers of children with disabilities? The research explores the mental health implications for parents of children with developmental disabilities, highlighting the increased risk of mental health issues such as depression, anxiety, and stress among these parents.

## 2 Materials and methods

The present study employs a qualitative research method to explore in-depth the perceptions of psychological problems resulting from having a child with a disability that lead to divorce, from the point of view of mothers of children with disabilities. Qualitative research is particularly effective in examining the subjective experiences and interpretations of individuals, providing a nuanced understanding of their thoughts, feelings, and motivations. This is essential for capturing the psychological problems resulting from having a child with a disability leading to divorce. The study adopts an inductive approach, emphasizing the centrality of themes emerging from the data to address research questions, avoiding predetermined themes by the researchers (Creswell et al., 2007; Ryder et al., 2019). To collect data, an open-ended technique was employed, as it captures rich perspectives, while theme development reveals patterns and insights into shared experiences (Braun and Clarke, 2006, 2022, 2023). This method aligns seamlessly with the inductive process, maintaining consistency with the comprehensive nature of the research inquiry. This inductive approach prioritizes themes arising from data, ensuring findings are driven by participants’ voices. Further details about participants, setting, and the methods of data analysis and collection will be elaborated in the following sections. The study is informed by previous research that highlights the psychosocial challenges experienced by female caregivers of children with disabilities, including stress, depression, anxiety, and isolation, which may lead to marital dissatisfaction and divorce (Risdal and Singer, 2004).

### 2.1 Sampling method and participants

In this research, a purposeful sampling technique was employed to recruit mothers offering insights into the experiences of divorce with a child attending a private day care center for people with disabilities in Saudi Arabia. The study was ethically reviewed and approved by the Standing Committee for Research Ethics at Hail University. All participants provided written informed consent.

#### 2.1.1 Inclusion criteria

The inclusion criteria were as follows: mothers of children with disabilities currently enrolled in the specific daycare center, who had experienced divorce, and demonstrated a willingness to participate in an interview.

#### 2.1.2 Exclusion criteria

Conversely, mothers of children with disabilities not enrolled in the daycare, those who were currently married or in a civil partnership, and mothers with a documented history of cognitive impairment or mental illness that could significantly impact their ability to participate were excluded from the study.

While contacting all divorced mothers with children using the daycare was attempted, a representative sample was ultimately chosen due to feasibility constraints (see Table 1 for a detailed overview of the sample demographics). This, combined with the sensitive nature of the topic, which can be emotionally challenging to discuss, resulted in a final sample size of five female participants. Moreover it’s important to note that qualitative research prioritizes in-depth exploration over statistical generalizability. Smaller sample sizes allow for richer data collection through in-depth interviews, enabling a deeper understanding of the participants’ experiences. In this study, the focus was on capturing the nuances of navigating divorce with a child with a disability, and the insights from five mothers provided valuable data for further exploration.

**Table 1:** Demographic characteristics of sample.

No.	Age	Employment status	Marital status	Socioeconomic status	Educational level	Years since divorce
1	38	Part-time	Divorced	Middle class	Bachelor	3
2	42	Unemployed	Divorced	Low-income	Graduate	5
3	53	Self-employed	Divorced	Upper middle class	Graduate	6
4	34	Full-time	Divorced	Working class	Graduate	4
5	45	Part-time	Divorced	Middle class	Graduate	4

Despite no quantitative data being collected on socioeconomic status (SES), participants were asked about their and their former spouse’s employment status and occupations during the interviews to build rapport and gain a broader understanding of their backgrounds. Throughout the study, participants were informed that their involvement was voluntary and could be discontinued at any time. They were also assured of the confidentiality of their responses. The selected sample had an average age of 34 and a range of 34 to 53, had an average of 4 years since their divorce, ranging from 4 to 8 years. Each of the female participants had one child with a disability.

### 2.2 Design and apparatus

The research instrument was a semi-structured, in-depth interview based on a list of eight questions, to obtain data from the participants regarding their perceptions and lived experiences (Van den Berg, 2005). There were follow-up questions based on the interviews’ directions. The list of 8 questions (see Appendix) was reviewed by two experts in special education who teach special education curriculum at a Saudi university. Based on the responses of these experts, two questions were eliminated, and four questions were adjusted to be more understandable to the proposed participant population. This resulted in the 8 questions that were incorporated into each interview. During the interviews, additional questions were asked based on the individual participant’s responses to obtain clarification or to encourage the participant to expand upon their answer. The interview questions focused primarily on obtaining an understanding of the participants’ views on Increasing Elementary school teachers’ Knowledge and Skills to Identify and Serve students with giftedness and learning disabilities in Saudi Arabia.

### 2.3 Data collection

The second investigator conducted personal interviews, utilizing face-to-face interactions with each participant. The study adopted the interview protocol inspired by the interview protocols in previous research studies like Bekker et al. (2019) and Helgeson et al. (2012) to ensure the questions fit the context of psychological problems resulting from having a child with a special needs/attention leading to divorce. Participants were asked a total of five questions. These interviews were conducted in private rooms within the care center, lasting approximately 30–46 min. The initial focus of the interviews was on gathering demographic details about mothers of children with disabilities and psychological problems, with the researcher delving deeper into the experiences and perspectives related to psychological problems. To ensure a comprehensive understanding, the researchers employed seven open-ended questions adopted with the study’s central theme in mind (Creswell et al., 2007). The interview process aimed to create an environment where participants could freely express their psychological problems. The researcher-initiated discussions by asking about the participants’ life and the child with disabilities and allowing participants to share their personal experiences after divorce. Additionally, as a primary focus of the study, questions were included to investigate the connection between life after divorce and having a child with a disability. Throughout the interviews, the researchers maintained a subtle approach, seeking additional information only when necessary for clarification or to expand the conversation. Supplementary prompts were used to summarize responses, confirm understanding, and encourage elaboration with specific examples. Following the interviews, participants were given the opportunity to share any additional thoughts or information they felt had not been previously addressed. For instance, the researcher may have paraphrased participants’ responses, saying, “From what I understand, you are saying…” and allowing participants to clarify or confirm their statements.

One key limitation faced by the researchers was the difficulty in recruiting participants for this sensitive and personal study. Many mothers were understandably hesitant to share their experiences, especially those related to divorce. To mitigate this, we built trust with the daycare center staff, who helped us approach potential participants in a sensitive manner. The interviews were conducted in private rooms at the daycare center, which provided a comfortable and familiar setting for the mothers.

### 2.4 Data analysis

The lead researcher on this study, wanted to address the issue of data saturation and the limitations team faced given the small sample size of 5 participants. Despite the challenges of recruiting divorced mothers of children with disabilities, researchers were able to achieve a level of data saturation that allowed us to comprehensively answer posed research questions.

The researchers utilized the thematic analysis method, as introduced by Braun and Clarke (2022), to analyze the collected interview data. This approach, known for its inherent flexibility, is frequently employed in qualitative research to ensure the accuracy and credibility of outcomes (Nowell et al., 2017). The flexibility of this method was particularly advantageous for this study and the interview process, enabling the researchers to meticulously and thoughtfully process the raw data.

During the data analysis process, researchers closely followed the thematic analysis method outlined by Braun and Clarke (2006), which involved six steps: (1) becoming familiar with the data, (2) producing initial codes, (3) identifying themes, (4) examining themes and underlying subthemes, (5) defining and titling themes, and (6) generating the report. They transcribed all interviews verbatim using audio recordings, and during the coding process, highlighted words or phrases representing a concept in each text copy and made notes in the side column. Researchers preserved the wording almost identical to the raw data’s phrasing, ensuring fidelity.

There were 76 codes recorded in a spreadsheet during the initial coding process of each transcription. Subsequently, the related data of these codes were analyzed by the researchers. For example, one of the 76 codes was “fear of the future,” applied when participants expressed concerns about their child’s long-term well-being. In one transcript, a participant said, “*I constantly worry about what will happen to my child when I’m gone. Who will take care of them*?” This was coded as “fear of the future” and grouped under the broader theme of “psychological strain.” Using qualitative analysis software, we tracked these codes to organize and identify key themes. This process allowed us to capture participants’ experiences while also revealing new insights, such as the subtheme of “religious acceptance.”

Following this, researchers held a meeting where they generated themes by analyzing and verifying the meaning of the codes. The research questions guided this code analysis, and irrelevant codes were highlighted in a specific color. The remaining codes relevant to the research questions were thoroughly analyzed to generate themes. In cases of disagreement among researchers regarding codes, the raw data was revisited. The analysis continued until we reached a consensus on codes and themes. The process was conducted in a formal and systematic manner to ensure the accuracy and credibility of the outcomes.

Despite the small sample size of 5 participants, researcher were able to achieve data saturation by the fourth interview, as they noticed recurring themes and a lack of new information emerging from subsequent interviews. To confirm this, researchers conducted a fifth interview, which reinforced their conclusion that the data collected was sufficient to answer the research questions.

## 3 Results

Through am iterative process, the researchers were able to select the data into two primary themes: “psychological strain” and “social issues.” Each of these main themes encompassed distinct subthemes that captured the nuanced experiences of the participants. For the “psychological strain” theme, the subthemes of “fear,” “shock,” and “religious acceptance” emerged. These subthemes vividly illustrated the profound emotional turmoil the mothers faced, as well as the role of religious faith in providing a sense of comfort and resilience. Similarly, the “social issues” theme was further delineated into the subthemes of “societal ridicule” and “absence of remarriage prospects.” These subthemes shed light on the significant social challenges the mothers encountered, including stigma, isolation, and the impact on their personal lives.

### 3.1 Psychological strain

The theme of psychological strain encapsulated the profound emotional and mental challenges faced by the mothers as they navigated the complexities of raising a child with a disability. This theme is further broken down into three subthemes, each highlighting a different aspect of the emotional burden these mothers experienced.

#### 3.1.1 Fear

The subtheme of fear captures the pervasive anxiety and dread that dominated the mothers’ thoughts. Their concerns extended beyond the present, as they worried deeply about the future of their children and the potential dissolution of their marital relationships. The uncertainty surrounding their child’s future care was a significant source of stress. One participant articulated this fear, saying, “*I constantly worry about what will happen to my child when I’m gone. Who will take care of them?*” This fear was not just about the child’s future but also about the impact of the child’s condition on their marriage. Another mother revealed, “*I was terrified that my husband would leave me because of our child’s disability. I did not know how I would cope on my own*.” The fear of abandonment compounded the stress, making the psychological strain even more intense.

#### 3.1.2 Shock

The subtheme of shock underscores the intense emotional impact that accompanied the initial diagnosis of their child’s disability. This shock often manifested as disbelief, denial, and a sense of disorientation as mothers grappled with the unexpected reality. One mother shared her experience: “*When the doctors told me my child had autism, I was completely devastated. I could not believe it was happening to us*.” This disbelief often delayed the acceptance process, as another participant mentioned, “*I was in denial for a long time, hoping it was just a phase. But as time went on, I had to face the reality of our situation*.” The shock was not just a momentary reaction but a prolonged state that hindered their ability to process and accept their new reality, further contributing to the psychological strain.

#### 3.1.3 Religious acceptance

In the midst of overwhelming psychological strain, some mothers found a refuge in their religious beliefs. The subtheme of religious acceptance highlights how faith provided them with a framework to make sense of their experiences, offering both emotional support and a sense of purpose. For many, religion became a vital coping mechanism, helping them to navigate the challenges of raising a child with a disability. One participant expressed, “*My faith in God has been the only thing that has kept me going. I know He has a plan, even if I do not understand it*.” This sentiment reflects a broader theme of surrendering to a higher power, which allowed mothers to find peace amid chaos. Another mother elaborated on how religious practices provided her with comfort: “*Praying and reading the Quran has helped me cope with the challenges of raising a child with a disability. It gives me a sense of peace*.” For these mothers, religion did more than offer comfort—it provided a structure that explained their child’s condition within a divine plan, granted them a sense of control in an uncontrollable situation, and thus relates to main theme of psychological strain, by being a coping mechanism. Religious acceptance, therefore, was not merely passive resignation but an active process of finding meaning and resilience in the face of profound psychological strain.

### 3.2 Social issues

The theme of social issues captures the profound impact that societal attitudes and stigmatization had on the participants’ lives. These mothers faced not only the emotional and logistical challenges of raising a child with a disability but also the harsh realities of societal judgment. This theme is explored through two subthemes: societal ridicule and the absence of remarriage prospects.

#### 3.2.1 Societal ridicule

The subtheme of societal ridicule delves deeply into the negative social interactions and judgment that mothers of children with disabilities endured within their communities. The stigma attached to having a child with a disability often led to overwhelming feelings of shame, isolation, and even self-doubt. One mother recounted her painful experiences: “*People in our neighborhood would stare and whisper whenever they saw my child. It made me feel so ashamed and isolated*. “The constant gaze and whispers created an emotional barrier, making it increasingly difficult for the mothers to participate in daily social life, further exacerbating their sense of alienation.

For some mothers, this judgment extended beyond the general public to their own families, magnifying the emotional toll. One mother revealed: “*Some family members even blamed me for my child’s disability, saying it was a punishment from God. It was so hurtful and unfair, especially coming from people I love*. “This narrative highlights the intersection of cultural and religious beliefs that often frame disability as a form of divine retribution, unfairly placing blame on the mothers for circumstances outside their control. Another mother shared: “*At family gatherings, no one would talk about my child, as if ignoring them would make the problem go away. It made me feel invisible, like my child and I did not matter*.” This sense of invisibility further deepened their emotional wounds.

The public scrutiny and judgment did not stop at stares and whispers. Some mothers reported being openly criticized for their parenting. One mother expressed: “*I’ve had neighbors tell me I wasn’t doing enough to ‘fix’ my child. They would suggest strange remedies, and when I declined, they would call me a bad mother*.” Another added: “*I was told that my child’s behavior was my fault because I did not discipline him enough. They did not understand that his disability wasn’t something I could just ‘correct’ with stricter parenting*. “.

The societal pressures also placed immense strain on their social support networks. As one mother explained: “*Friends stopped inviting me to social gatherings because they thought my child would ‘ruin’ the event. I lost so many friendships because people did not want to deal with the ‘inconvenience’ of my child’s condition*.” The erosion of these support systems left many mothers feeling utterly alone in their struggles.

In some cases, the community’s religious framing of disability intensified the mothers’ feelings of guilt and helplessness. “*I was told that my child’s disability was a punishment for my past sins*,” one mother disclosed. “*It was devastating to hear, especially because I was already questioning myself so much*.” This constant barrage of societal ridicule not only strained their mental health but also left them grappling with a sense of unworthiness, both as individuals and as mothers.

Ultimately, the societal and familial judgments they faced eroded their confidence and well-being. Another mother reflected: “*Over time, I started to believe what people were saying—that I was a failure, that I had somehow caused my child’s condition. It took me a long time to realize that none of this was my fault*.” For many, the weight of societal ridicule became an additional burden, making their already challenging journey even more isolating and painful”.

#### 3.2.2 Absence of remarriage prospects

The subtheme of the absence of remarriage prospects illustrates how societal expectations and stigma affected the personal lives of these mothers, particularly their chances of forming new romantic relationships after divorce or widowhood. The participants expressed deep concerns about how their child’s disability diminished their desirability as potential partners. As one mother shared, “*After my divorce, I was told that no one would want to marry me because of my child’s special needs. It felt like my life was over*.” This statement reflects the harsh reality that these mothers faced—being judged not only for their marital status but also for their responsibilities as caregivers. Another participant added, “*I’ve given up on the idea of finding a new partner. The stigma and societal pressure are just too much to bear*.” The absence of remarriage prospects was not just about personal rejection but also about the societal pressures that dictated their worthiness as potential spouses. This pressure often led to the abandonment of personal aspirations and a sense of hopelessness regarding their future.

The findings from this study highlight the multifaceted challenges faced by Saudi mothers of children with disabilities, underscoring the need for comprehensive support systems and interventions to address their psychological and social well-being.

## 4 Discussion

The present qualitative study aimed to explore the psychological challenges faced by Saudi mothers raising children with disabilities and how these challenges might have contributed to the risk of divorce. The findings provide valuable insights into the unique experiences of this population, while also highlighting areas of convergence with previous research conducted in other cultural contexts.

The first theme that emerged from the interviews with the Saudi mothers highlighted the profound emotional turmoil they experienced upon realizing their child’s disability. This finding aligns closely with previous research on the negative psychological impact that often accompanies a child’s disability diagnosis. Studies have consistently shown that the initial period after receiving a child’s disability diagnosis can be a profoundly distressing time for parents. Al-Shammari and Al-Harbi's (2021) research on Saudi families found that parents, especially mothers, frequently grapple with intense feelings of fear, shock, and grief during this critical transition. Similarly, Pasalic's (2023) work has emphasized the significant emotional strain parents face as they come to terms with their child’s condition and its implications for the family’s future.

The subtheme of ‘fear’ that the researchers identified further underscores the worries and anxieties that consumed these mothers. Prior literature has documented how parents of children with disabilities often agonize over their child’s long-term prospects and the potential impact on their marital relationship (Haimour and Abu-Hawwash, 2012; Risdal and Singer, 2004). The Saudi mothers in this study echoed these concerns, expressing deep-seated fears about their child’s wellbeing and the stability of their marriages.

An interesting feature of this research’s finding is the subtheme of “religious acceptance” which highlighted a culturally unique coping mechanism for these Saudi mothers. While prior literature has emphasized religion as a primary coping strategy in religious communities (Karaca and Şener, 2019), the present study found that the mothers’ faith in God and religious practices served as a protective factor, providing them with a sense of peace and resilience amidst the challenges they faced.

This alignment between the current study’s findings and the broader body of research on the psychological challenges faced by parents of children with disabilities lends credibility to the researchers’ analysis. It is obvious that psychological strain and distress experienced by these mothers can indeed become a significant cause of divorce. The constant fear, shock, and distress they face can lead to marital dissatisfaction and ultimately, divorce. This is also supported by previous research indicating that the psychological problems associated with having a child with a disability can contribute to marital instability and the breakdown of family relationships (Al-Shammari and Al-Harbi, 2021; Namkung et al., 2015).

Moreover, the study found that the societal stigma and ridicule faced by these mothers further exacerbate their psychological strain (Gray, 2002; Woodgate et al., 2008). The social isolation and negative societal attitudes towards disability can intensify the emotional distress and anxiety experienced by these mothers, leading to increased marital tensions and a higher risk of divorce (Çaynak et al., 2022; Čolić et al., 2022).

Iudici et al. (2019) provide further context on societal attitudes by examining how different community roles construct and discuss disability. Their findings reveal that those closest to people with disabilities often view disability more narrowly, while professionals take a more generative approach. This highlights the need for more inclusive discussions around disability, as the narrow views held by some community members can perpetuate stigma and isolation, which in turn exacerbates the psychological strain on mothers.

The main theme social issues and its subthemes shed light on critical issues not extensively explored in Saudi society thus far. Previous work has documented the social stigma and isolation faced by families of individuals with disabilities globally (Çaynak et al., 2022; Čolić et al., 2022). However, the present research offers context-specific insights into the ridicule, shame, and frustration experienced by Saudi mothers due to harmful societal attitudes in their local communities. The subtheme ‘absence of remarriage prospects’ also highlighted a novel challenge, considering remarriage norms in the region.

Adding to the complexity of these social issues are the broader societal factors affecting women in Saudi Arabia. Zamberi Ahmad (2011) discusses the paradox between the country’s commitments to modernity and education and its adherence to religious purity, especially regarding women’s roles. This tension can exacerbate the challenges faced by mothers of children with disabilities, as societal expectations around gender and family roles may limit their opportunities for support and empowerment. Al-Rasheed (2013) provides historical context on the role of women in Saudi Arabia, noting that early goals of women’s education were primarily focused on producing teachers and wives, rather than active citizens. Although policies have since evolved to encourage education and careers for women, the lingering effects of these early goals may still influence societal attitudes toward divorced mothers and their remarriage prospects.

Smith and Abouammoh (2013) also discuss the rapid growth of female enrollments in higher education in Saudi Arabia but raise concerns about the quality and range of opportunities available, particularly in fields needed for the job market. This suggests that despite progress in education, systemic barriers remain, which may further limit the support and opportunities available to mothers of children with disabilities.

The findings of this study suggest that divorced Saudi mothers raising children with disabilities faced significant psychological strain and social challenges. While the research itself did not explicitly discuss avoidance as a coping mechanism, the themes that emerged offer clues about how these mothers might have responded to the immense difficulties they encountered.

This intense emotional turmoil, coupled with societal ridicule (a known risk factor for avoidance coping), suggests that some mothers might have turned to avoidance strategies (Carver et al., 1989; Folkman and Moskowitz, 2004). Avoidance, in this context, could involve withdrawing from social interactions or neglecting aspects of their lives to temporarily alleviate distress. This, in turn, could increase the risk of divorce. Further supporting this notion, social exchange theory suggests individuals evaluate the costs and benefits of relationships before making decisions (Ahmad et al., 2022). In this study, the psychological burdens associated with raising a child with a disability could be perceived as costs, while the benefits might include the child’s love and the fulfillment of parenthood. If the perceived costs outweigh the benefits, as they might in some cases, divorce becomes a possible outcome.

### 4.1 Practical implications, limitations and suggestions

This study highlights the need for tailored support for divorced Saudi mothers raising children with disabilities. Specialized clinics and support groups can address their intense emotional strain through mental health services and coping strategies. Public awareness campaigns and policy changes can combat societal ridicule and isolation, fostering a more inclusive environment. Support should be designed with Saudi Arabia’s cultural and religious context in mind, potentially incorporating religious teachings to enhance program effectiveness. Increased funding for specialized services and anti-discrimination laws are crucial policy changes to support these families. Future research can explore coping mechanisms, socioeconomic factors, and long-term impacts of divorce, while comparative studies across regions or cultures could offer further insights. By addressing these needs, we can create more effective support systems and enhance the well-being of these families.

It is important to acknowledge the potential biases and limitations of this study. The researchers’ own biases and assumptions could have influenced the data collection and analysis processes, potentially shaping the themes that emerged. For example, the researchers’ own cultural backgrounds and experiences may have led them to emphasize certain aspects of the participants’ narratives over others. Additionally, the recruitment of participants solely from a private daycare center may have excluded the perspectives of mothers from different socioeconomic or educational backgrounds, potentially missing important nuances in their experiences. Furthermore, the reliance on self-reported data from the participants introduces the possibility of recall bias or social desirability bias, where participants may have selectively shared or omitted information based on their perceptions of what the researchers wanted to hear. Future research should employ multiple researchers from varied backgrounds and conducting member checking procedures could also help mitigate potential biases and strengthen the credibility of the study. Additionally, the researchers could have explored the potential influence of factors such as the participants’ socioeconomic status, level of education, and duration of divorce on their experiences and coping strategies. Examining these variables could provide a more comprehensive understanding of the challenges faced by this population. Despite these limitations, the present study contributes to the growing body of literature on the psychological challenges faced by families of children with disabilities, particularly in the context of Saudi Arabian culture. The findings underscore the need for tailored support systems and interventions to address the unique needs of these families, ultimately enhancing their overall well-being and resilience.

## 5 Conclusion

This study explored the psychological challenges faced by 5 Saudi mothers of children with disabilities. The interviews revealed two overarching themes—Psychological Strain (with subthemes of Fear, Shock and Religious Acceptance) and Social Problems (with subthemes of Societal Ridicule and Absence of Remarriage Prospects). The findings provide valuable perspectives on the emotional distress and social issues experienced by these mothers within the Saudi cultural context. The results enhance understanding of the challenges faced by families in this situation.

## Data Availability

The raw data supporting the conclusions of this article will be made available by the authors, without undue reservation.

## Appendix

**Interview questions**

1. How has the experience of raising a child with a disability affected your emotional well-being and mental health?
2. Can you describe the specific challenges and stressors you have faced in caring for a child with a disability, and how these have impacted your relationship with your spouse?
3. In what ways has the care of your child with a disability influenced your overall quality of life and daily functioning?
4. How have you coped with the psychological burden of caring for a child with a disability, and what support systems have been most helpful to you in managing this pressure?
5. Can you share any specific instances or situations that have put a strain on your marriage or led to increased stress due to the challenges of raising a child with a disability?

## References

[ref1] Ahmad R. Nawaz M. R. Ishaq M. I. Khan M. M. Ashraf H. A. (2022). Social exchange theory: systematic review and future directions. Front. Psychol. 13. doi: 10.3389/fpsyg.2022.1015921, PMID: 36710813 PMC9878386

[ref001] Al-Maamari W. (2015). Reasons leading to divorce from the point of view of divorced and divorced women in Omani society. Journal of the Arab American Academy of Science and Technology, USA, 6, 19.

[ref4] Al-Rasheed M. (2013). A most masculine state: Gender, politics and religion in Saudi Arabia: Cambridge University Press.

[ref5] Al-Shammari N. (2015). “Autism” and “disability” raise the divorce rate among Saudis. Available at: https://www.aleqt.com/2013/12/26/article_810459.html.

[ref6] Al-Shammari Y. S. Al-Harbi N. H. H. (2021). Divorce in the Kingdom of Saudi Arabia, the development of its rates, characteristics, and geographical variation. J. Center Geograph. Cartograph. Res. 18, 311–355.

[ref8] Alwhaibi R. M. Zaidi U. Alzeiby I. Alhusaini A. (2018). Quality of life and socioeconomic status: a comparative study among mothers of children with and without disabilities in Saudi Arabia. Child Care Pract. 26, 62–80. doi: 10.1080/13575279.2018.1512951

[ref9] Anderson J. (2014). The impact of family structure on the health of children: effects of divorce. Linacre Q. 81, 378–387. doi: 10.1179/0024363914z.00000000087 25473135 PMC4240051

[ref10] Bekker C. I. Deacon E. Segal D. (2019). Meaning in life experienced by parents of children living with diabetes. Health Psychol. Open 6:2055102919832221. doi: 10.1177/2055102919832221, PMID: 30858981 PMC6402055

[ref11] Braun V. Clarke V. (2006). Using thematic analysis in psychology. Qual. Res. Psychol. 3, 77–101. doi: 10.1191/1478088706qp063oa

[ref12] Braun V. Clarke V. (2022). Conceptual and design thinking for thematic analysis. Qual. Psychol. 9, 3–26. doi: 10.1037/qup0000196

[ref13] Braun V. Clarke V. (2023). Toward good practice in thematic analysis: avoiding common problems and be(com)ing a knowing researcher. Int. J. Transgender Health 24, 1–6. doi: 10.1080/26895269.2022.2129597, PMID: 36713144 PMC9879167

[ref15] Carver C. S. Scheier M. F. Weintraub J. K. (1989). Assessing coping strategies: a theoretically based approach. J. Pers. Soc. Psychol. 56, 267–283. doi: 10.1037/0022-3514.56.2.267 2926629

[ref16] Çaynak S. Özer Z. Keser İ. (2022). Stigma for disabled individuals and their family: a systematic review. Perspect. Psychiatr. Care 58, 1190–1199. doi: 10.1111/ppc.12893, PMID: 34121194

[ref17] Čolić M. Dababnah S. Milačić-Vidojević I. (2022). A model of internalized stigma in parents of individuals with disabilities. Int. J. Dev. Disabil. 68, 924–932. doi: 10.1080/20473869.2021.1924032, PMID: 36568618 PMC9788690

[ref18] Creswell J. W. Hanson W. E. Clark Plano V. L. Morales A. (2007). Qualitative research designs. Couns. Psychol. 35, 236–264. doi: 10.1177/0011000006287390

[ref19] Daroni G. A. Salim A. Sunardi S. (2018). Impact of parent’s divorce on children’s education for disability kids. Indonesian J. Dis. Stud. 5, 1–9. doi: 10.21776/ub.IJDS.2018.005.01.1

[ref20] Doherty S. (2008). Arrested development the day-to-day struggles of autistic children affect entire family. The capital Times, 25.

[ref21] Folkman S. Moskowitz J. T. (2004). Coping: pitfalls and promise. Annu. Rev. Psychol. 55, 745–774. doi: 10.1146/annurev.psych.55.090902.141456 14744233

[ref22] Gray D. E. (2002). Everybody just freezes. Everybody is just embarrassed”: felt and enacted stigma among parents of children with high functioning autism. Sociol. Health Illn. 24, 734–749. doi: 10.1111/1467-9566.00316

[ref23] Gruszka P. Ganahl K. Stasch N. Burger C. Haberlandt E. Bauer S. M. (2023). Parental anxiety and depression are associated with adverse mental health in children with special needs during the COVID-19 pandemic. Front. Public Health 11:1254277. doi: 10.3389/fpubh.2023.1254277, PMID: 38074710 PMC10699309

[ref24] Haimour A. I. Abu-Hawwash R. M. (2012). Evaluating quality of life of parents having a child with disability. Int. Interdiscipl. J. Educ. 1, 37–43.

[ref25] Hammad M. A. (2021). The impact of the COVID-19 crisis on the role of mothers towards their disabilities children. Int. J. Soc. Sci. Hum. Res. 4, 3297–3304. doi: 10.47191/ijsshr/v4-i11-32

[ref26] Helgeson V. S. Becker D. Escobar O. Siminerio L. (2012). Families with children with diabetes: implications of parent stress for parent and child health. J. Pediatr. Psychol. 37, 467–478. doi: 10.1093/jpepsy/jsr110, PMID: 22267104 PMC3334535

[ref27] Iudici A. Antonello A. Turchi G. (2019). Intimate partner violence against disabled persons: clinical and health impact, intersections, issues and intervention strategies. Sex. Cult. 23, 684–704. doi: 10.1007/s12119-018-9570-y

[ref28] Karaca A. Şener D. K. (2019). Spirituality as a coping method for mothers of children with developmental disabilities. Int. J. Dev. Disabil. 67, 112–120. doi: 10.1080/20473869.2019.1603730, PMID: 34141404 PMC8115452

[ref30] Madi S. M. Mandy A. Aranda K. (2019). The perception of disability among mothers living with a child with cerebral palsy in Saudi Arabia. Glob. Qual. Nurs. Res. 6:233339361984409. doi: 10.1177/2333393619844096, PMID: 31065572 PMC6488775

[ref002] Mursi N. B. Sulaimani M. F. (2022). Influence of context related factors on saudi special education teachers’ understanding of evidence, evidence-based, and evidence-based practices. Problems of Education in the 21st Century, 80, 588–601. doi: 10.33225/pec/22.80.588, PMID: 34141404

[ref32] Namkung E. H. Song J. Greenberg J. S. Mailick M. R. Floyd F. J. (2015). The relative risk of divorce in parents of children with developmental disabilities: impacts of lifelong parenting. Am. J. Intellect. Dev. Disabil. 120, 514–526. doi: 10.1352/1944-7558-120.6.514, PMID: 26505872 PMC4624231

[ref33] Nowell L. S. Norris J. M. White D. E. Moules N. J. (2017). Thematic analysis: striving to meet the trustworthiness criteria. Int J Qual Methods 16:160940691773384. doi: 10.1177/1609406917733847

[ref34] Pasalic A. (2023). The importance of early intervention focused on parent training for preschool-aged children with autism: a systematic literature review. Multidisciplinarni Pristupi U Edukaciji I Rehabilitaciji 5, 39–72. doi: 10.59519/mper5204

[ref35] Risdal D. Singer G. H. (2004). Marital adjustment in parents of children with disabilities: a historical review and meta-analysis. Res. Pract. Persons Severe Disabil. 29, 95–103. doi: 10.2511/rpsd.29.2.95

[ref36] Ryder M. Jacob E. Hendricks J. (2019). An inductive qualitative approach to explore nurse practitioners views on leadership and research: an international perspective. J. Clin. Nurs. 28, 2644–2658. doi: 10.1111/jocn.14853, PMID: 30916828

[ref37] Salehi F. Raji P. Mahmoodian M. Dadgar H. Baghestani A. R. (2017). Quality of life of mothers of children with autism spectrum disorders and its relationship with severity of disorder and child’s occupational performance. J. Modern Rehabil. 11, 167–174.

[ref38] Sharqi R. (2018). Social stigma of divorced women: a socio-anthropological analysis. J. Hum. Soc. Sci. 22, 171–180.

[ref39] Smith L. Abouammoh A. (2013). Higher education in Saudi Arabia: achievements, challenges and opportunities. Netherlands: Springer.

[ref40] Song J. Mailick M. R. Greenberg J. S. (2018). Health of parents of individuals with developmental disorders or mental health problems: impacts of stigma. Soc. Sci. Med. 217, 152–158. doi: 10.1016/j.socscimed.2018.09.044, PMID: 30333078 PMC6202233

[ref003] Van den Berg A. E. ter Heijne M. (2005). Fear versus fascination: An exploration of emotional responses to natural threats. Journal of Environmental Psychology, 25, 261–272. doi: 10.1016/j.jenvp.2005.08.004

[ref41] Woodgate R. L. Ateah C. Secco L. (2008). Living in a world of our own: the experience of parents who have a child with autism. Qual. Health Res. 18, 1075–1083. doi: 10.1177/1049732308320112, PMID: 18650563

[ref42] Zamberi Ahmad S. (2011). Businesswomen in the kingdom of Saudi Arabia. Equal. Divers. Inclus. Int. J. 30, 610–614. doi: 10.1108/02610151111167052

